# Translating Ecological Realism into Ecotoxicology: An Experimental Approach for Assessing Plant Biodiversity

**DOI:** 10.1021/acs.est.6c06092

**Published:** 2026-09-19

**Authors:** Rosa Kim, Kwang Hyun Hwang, Hi Gyu Moon, Shin Woong Kim

**Affiliations:** 675021 Korea Institute of Toxicology, 17 Jegok-gil, Jinju 52834, Republic of Korea

**Keywords:** cadmium, grassland, network, soil seed bank, toxicity

## Abstract

Traditional ecotoxicological assessments predominantly rely on single-species bioassays, often failing to address the complex interspecific interactions and structural resilience inherent in natural ecosystems. In this study, we developed a community-level ecotoxicological approach using soil seed banks as a representative ecological proxy. By reconstructing diverse plant assemblages directly from field-sampled soil (26 species, 455 individual plants), we evaluated the impacts of a model chemical (cadmium, Cd) on multidimensional end points, including plant diversity, biomass productivity, and co-occurrence networks. Our findings revealed a discrepancy between individual and community-level sensitivities: the effective concentration 10% (EC_10_) for the Shannon–Wiener index was 76.36 mg kg^–1^, which is approximately 20-fold higher than the hazardous concentration 5% derived from conventional species sensitivity distribution (SSD) models. Although these metrics differ in their conceptual basis, this gap offers critical insights into the conservative margin of regulatory standards. While total community biomass significantly declined at high Cd concentrations, dominant species exhibited robust tolerance, suggesting a selective filtration of sensitive species rather than a uniform inhibition across all constituents. Furthermore, co-occurrence network analysis demonstrated that Cd stress reduced structural complexity while simultaneously increasing interspecific co-occurrence strength among survivors. These results demonstrate that plant communities possess a significantly higher buffering capacity against chemical stress. Our approach provides an ecologically realistic platform for the next generation of ecological risk assessments, offering deeper and more direct insights into anthropogenic impacts on terrestrial biodiversity and ecosystem stability.

## Graphical abstract

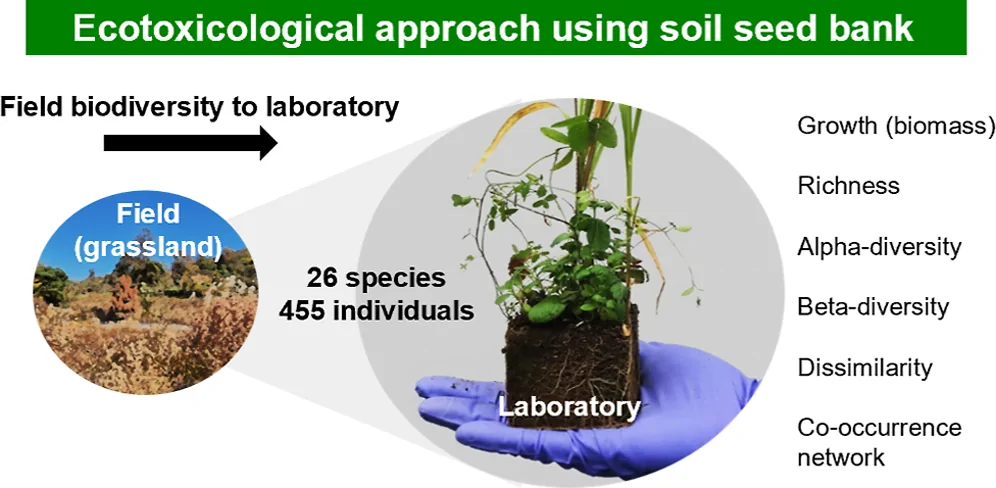

## 1 Introduction

Ecotoxicology aims to protect biodiversity and ecosystem services against the structural and functional damages induced by anthropogenic chemical stress.^1^ As a cornerstone of ecological risk assessment, the species sensitivity distribution (SSD) model provides a statistically rigorous framework for quantifying toxicity variation across multiple trophic levels, thereby facilitating the derivation of protective thresholds.^2,3^ However, this framework is often constrained by its reliance on single-species laboratory bioassays,^4,5^ which fundamentally overlook the interspecific interactions inherent in natural ecosystems.^6^ While scaling ecotoxicological perspectives from individual to community levels enhances ecological realism, the difficulty of data acquisition commensurately increases due to higher complexity and lower reproducibility.^7^ Although mesocosm and microcosm studies offer valuable insights into ecosystem-level dynamics,^8^ these designs are often limited by high costs and logistical complexity.^9^ Furthermore, field-scale experiments frequently struggle to isolate specific environmental drivers from observed effects.^10,11^ Consequently, ecotoxicological impacts at the community level remain relatively understudied.

This limitation is particularly pronounced in plant ecotoxicology. Standardized plant toxicity tests, such as ISO or OECD protocols, typically utilize a restricted set of model species (e.g., *Lactuca sativa* or *Avena sativa*).^12,13^ While these assays are efficient for chemical hazard screening, they fail to capture critical community-level dynamics.^14^ In natural plant communities, species engage in a myriad of positive and negative interactions, occurring directly through facilitation and competition, or indirectly via alterations in abiotic and biotic conditions.^15^ However, characterizing these comprehensive interactions remains a methodological challenge due to the vast number of potential pairwise combinations across various time scales.^14^ Rather than addressing the plant community dynamics directly, recent research has frequently focused on soil microbial communities associated with specific plant types, such as metal-tolerant species.^16,17^ Although efforts to predict plant biodiversity changes have employed ecological modeling with field monitoring (e.g., landscape ecotoxicology),^11,18^ these predictive tools often rely on static snapshots of standing vegetation. While field-based observations offer high ecological realism, they frequently struggle to define clear dose–response relationships or to establish reliable ecological baselines (e.g., reference site). Despite the evolution of ecotoxicological methodologies, the gap between highly controlled laboratory bioassays and the multidimensional reality of field environments persists.^1,5^

This study presents an experimental approach for assessing plant community responses to chemical stress under laboratory conditions. We employed the concept of the soil seed bank, defined as the natural reservoir of dormant seeds within the soil profile.^19,20^ The seed bank represents the regenerative potential and historical genetic diversity of a site.^20^ By utilizing the soil seed bank as a target plant community, we designed the experiment to incorporate the natural species pool and soil matrix into a controlled laboratory setting. Field soil was collected in late autumn from a local grassland to capture peak seed density and high germination potential, providing a representative matrix for assessing community assembly.^21^ The soil was subsequently exposed to cadmium (Cd), selected as a model contaminant due to its environmental persistence, high bioavailability, and well-documented toxicity. The activation of dormant seed banks under optimized, spring-like conditions (controlled thermal and moisture regimes) led to the emergence of 26 species (455 individuals). This approach facilitated the comprehensive quantification of community-level diversity and species composition. We hypothesize that (1) our method for seed bank activation yields an ecologically relevant and laboratory-scale plant community, (2) such field-derived assemblages exhibit sufficient sensitivity to chemical stress, and (3) community-level metrics offer more integrated ecological insights compared to traditional single-species assays.

## 2 Materials and Methods

### 2.1 Soil Collection and Treatment

Bulk soil was collected in November 2025 from a natural grassland at Korea Institute of Toxicology (Jinju, South Korea). The sampling site is characterized by a diverse temperate plant community, dominated by perennial grasses and various forbs that contribute to a robust and heterogeneous soil seed bank. To capture the maximum potential of the regenerative species pool, a 3 × 3 m quadrat was randomly established, and pretreated by removing aboveground vegetation in preparation for the winter dormancy period. The soil surface was then carefully leveled to expose the uppermost mineral horizon, and approximately 50 kg of soil was sampled from the topsoil (0–10 cm depth). The soil was air-dried at low temperatures (5–10 °C) under dark conditions and subsequently passed through a 4 mm sieve. To ensure rigorous homogenization, 20 kg of the sieved soil was partitioned into ten 2 kg subunits, each of which was mixed manually for 5 min. These subunits were further divided into 200 g aliquots and consolidated into a single bulk container using a stepwise layering and homogenization process to achieve uniform spatial distribution. For the experimental units, 180 g of the homogenized soil (water holding capacity: 70% w/w; pH: 6.6) was placed into individual plastic pots (75 × 75 × 100 mm) and stored at room temperature in the dark.

To prepare the loading soil for Cd treatment, a subset of the soil was sterilized at 121 °C for 20 min. This sterilization step was conducted to mitigate localized microbial shocks caused by concentrated chemical exposure.^22^ Twenty g of this sterilized loading soil was placed in a plastic cup, to which 10 mL of CdCl_2_ (Sigma-Aldrich) solution was added to reach test concentrations. After the loading soils were air-dried, they were thoroughly homogenized with 180 g of the base soil in each pot. Finally, distilled water was added to adjust the soil moisture content to 60% of the water holding capacity. Soil pH and electrical conductivity (1:5 w/v) were measured across all concentrations using a multiparameter meter (Multi 9630 IDS, WTW, Weilheim, Germany). Total and bioavailable Cd concentrations were quantified using inductively coupled plasma-optical emission spectrometry (ICP-OES; 5800, Agilent, USA). Total concentrations were quantified after microwave-assisted acid digestion (HNO_3_/H_2_O_2_), whereas bioavailable Cd was extracted using 0.01 M CaCl_2_ solution (1:10 w/v, shaken for 2 h).^23^ Across all Cd treatment levels, soil pH remained stable between 6.50 and 6.62, and EC values remained consistently low, ranging from 0.08 to 0.16 dS m^–1^. The total nominal (measured) Cd concentrations were 0 (not detected, *n* = 18), 1 (2.37), 5 (7.84), 10 (24.17), 50 (76.07), and 100 (140.65) (*n* = 10 per treatment) mg Cd kg^–1^ dry soil, and 0.01 M CaCl_2_-extractable (bioavailable) Cd concentrations ranged from 0.004 to 0.706 mg L^–1^ (Table S1).

### 2.2 Plant Community Toxicity Tests

This study employs an experimental approach to investigate the toxicological effects on the plant community, encompassing diversity, species composition, and network structures (Figure 1). To achieve this, each pot was incubated in a growth room (22 °C, 60% relative humidity, 16 h photoperiod), with soil moisture maintained at 60% of the water-holding capacity throughout the experimental period. These conditions simulated a spring-like environment to facilitate the germination and activation of the indigenous soil seed bank. After an incubation period of 49 days, all 455 individual plants were manually harvested. The fresh biomass of shoots and roots was measured for each individual, after which every specimen was identified to the species level and enumerated to assess community-level responses across all treatments.

**Figure 1:**
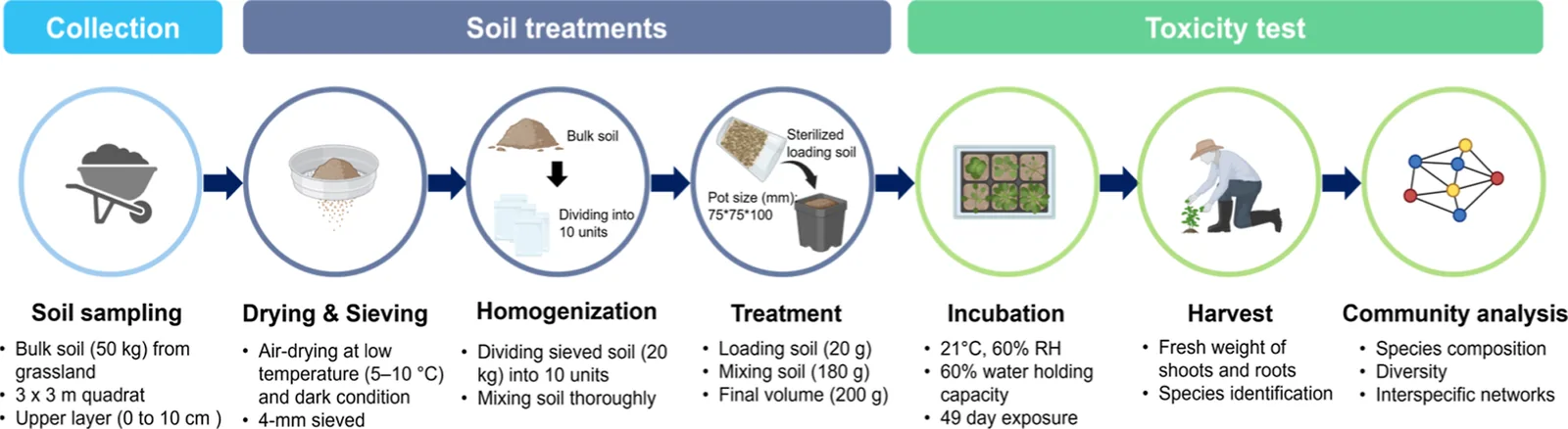
Schematic workflow of our experimental approach using soil seed bank. The process consists of three main stages: (i) collection of bulk soil samples; (ii) soil treatments including sieving, homogenization, and pot preparation; and (iii) plant community toxicity test involving plant cultivation, phenotypic observation, and data analysis.

### 2.3 Identification of Plant Species

Following the harvest, we initially performed a visual sorting of the 455 individual plants into 30 putative morphospecies. To ensure taxonomic accuracy, we subsequently conducted DNA barcoding, which finalized the identification of 26 distinct species (Table S2). All voucher specimens have been deposited at the Korea Institute of Toxicology (Jinju, South Korea) for future reference. Genomic DNA (gDNA) was extracted from 10–80 mg of plant tissue using the HiGene Genomic DNA Prep Kit for Plant Tissue (BioFACT, South Korea). Samples were ground into a fine powder in liquid nitrogen and lysed in a lysis buffer containing Proteinase K (20 mg mL^–1^) and RNase A (4 mg mL^–1^) at 65 °C for 10 min. Following centrifugation at 13,000 rpm for 5 min, the supernatant was mixed with binding buffer, and the resulting mixture was purified via spin-column chromatography followed by washing with 80% ethanol and washing buffer. The gDNA was eluted in 50 μL of DNA hydration solution and stored at −20 °C.

For species identification, the internal transcribed spacer (*ITS*) and *rbcL* regions were amplified using universal primers.^24,25^ PCR was performed in a 25 μL reaction volume containing 2 μL of template gDNA, 12.5 μL of BioFACT Lamp Taq Master mixture (DNA polymerase, BioFACT, South Korea), 1 μL each of forward and reverse primers (10 μM), and distilled water up to the final volume. The amplification was carried out using the following thermal cycling conditions: initial denaturation at 95 °C for 2 min; 32 cycles of denaturation at 95 °C for 20 s, annealing at 56 °C (*ITS*) or 53 °C (*rbcL*) for 40 s, and extension at 72 °C for 1 min; final extension at 72 °C for 5 min. PCR products were purified using the HiGene Gel & PCR Purification System (BioFACT, South Korea) and visualized via agarose gel electrophoresis to confirm quality and concentration. The amplicon library was sequenced using the ABI BigDye Terminator v3.1 Cycle Sequencing Kit (Applied Biosystems, CA, USA). The acquired nucleotide sequences were processed through the ABI 3730XL DNA Analyzer (Applied Biosystems, CA, USA) to generate consensus sequences. We subjected the resulting sequences to BLASTn searches of the NCBI GenBank database and assigned taxonomies based on a minimum identity of 99% (Table S2). The morphological traits were cross-referenced.

### 2.4 Statistical Analysis

All statistical analyses and visualizations were performed in R (version 4.5.1). Normality and homogeneity of univariate parameters (including number of plants, richness, diversity indices, and biomass data) were verified using the Shapiro–Wilk and Bartlett’s tests, respectively.^26,27^ To identify significant differences between the control and each treatment group, Dunnett’s posthoc test was employed using the *multcomp* package.^28^ When the assumptions for parametric analysis (Shapiro–Wilk and Bartlett’s tests) were not met (*p* < 0.05), the nonparametric Kruskal–Wallis test was applied, followed by Dunn’s posthoc test with Holm’s adjustment for comparisons against the control group (*rstatix* package, Table S3). Statistical significance was defined at *p* < 0.05, with levels of significance denoted as follows: * (*p* < 0.05), ** (*p* < 0.01), and *** (*p* < 0.001). All plots were generated using the *ggplot2* package.^29^

To evaluate shifts in species composition across treatments, community data (shoot biomass per species) were square-root transformed (Hellinger transformation) to reduce the influence of highly dominant species. Nonmetric multidimensional scaling (NMDS) based on Bray–Curtis dissimilarity was performed using the *vegan* package to visualize differences in community structure. Significant differences in community composition among treatment groups were assessed using Permutational Multivariate Analysis of Variance (PERMANOVA) with 9999 permutations via the *adonis2* function, and pseudo-F statistic, associated degrees of freedom, the coefficients of determination (R^2^), and *p* value were determined.

The relationships between alpha-diversity indices (Shannon–Wiener index, Simpson index) and total shoot biomass were evaluated using Pearson correlation and linear regression analyses. Analysis of Covariance (ANCOVA) was subsequently performed to compare regression slopes across treatment groups, ensuring the assumption of homogeneity of regression slopes was met. Regression models were fitted for both the overall community and specific target species within individual treatment groups to determine the coefficients of determination (*R* ^2^) and *p* value. All linear modeling and covariate analyses were conducted using the *stats* package.

To characterize the interspecific co-occurrence and community stability, network analysis was performed across each treatment. The network was constructed and visualized using a circular layout via the *tidygraph* and *ggraph* packages.^30^ Interspecific interaction strengths were quantified using Spearman’s rank correlation coefficients (*r*_s_) calculated from the shoot biomass of each species. Co-occurrence networks were constructed using a dual-filtering strategy. Edges were retained only if they satisfied both a minimum correlation magnitude threshold (|*r*_s_| ≥ 0.3) and statistical significance (*p* < 0.1).^31,32^ This approach preserves fundamental system topology while balancing statistical power under small sample sizes inherent to pot experiments.^31,32^ Additionally, self-correlations, loops, and multiple edges were systematically removed. The topological properties were characterized by calculating edge density, mean edge weight (interspecific association strength), average strength (overall node connectedness), and modularity using the *igraph* package.^33^ Furthermore, to control for potential network density biases induced by variations in the nodes across treatments, the minimum observed number of nodes was selected as the baseline target node size. For each treatment group, 12 species were randomly subsampled without replacement from the species pool, and a subnetwork was reconstructed to compute its network density. This subsampling procedure was repeated iteratively (1000 permutations) using the *purrr* and *dplyr* packages to generate a standardized empirical density distribution for each treatment.

## 3 Results and Discussion

### 3.1 Effects on Plant Community Diversity and Structure

The number of individuals per pot significantly decreased (Figure 2A) at 140.65 mg kg^–1^ of Cd concentrations. Concurrently, species richness (number of species) and the Shannon–Wiener index significantly declined at the highest concentration, whereas the Simpson index increased at this concentration (Figure 2B–D). The plant community was dominated by eight species: *Setaria viridis* (22%), *Oxalis stricta* (22%), *Erigeron philadelphicus* (13%), *Artemisia stolonifera* (11%), *Veronica persica* (5%), *Vicia hirsuta* (4%), *Ipomoea hederacea* (3%), *Corchoropsis tomentosa* (2%), with 18 additional species comprising the remaining 18% (Figure 2E; Table S4 and S5). Native and invasive species accounted for 67% and 32% of the total, respectively (Figure 2F). Despite the shifts in alpha-diversity, NMDS ordination indicated that overall community composition remained relatively resilient across the Cd treatments (*F*_1,66_ = 1.577, *R* ^2^ = 0.023, *p* = 0.119) (Figure 2G).

**Figure 2:**
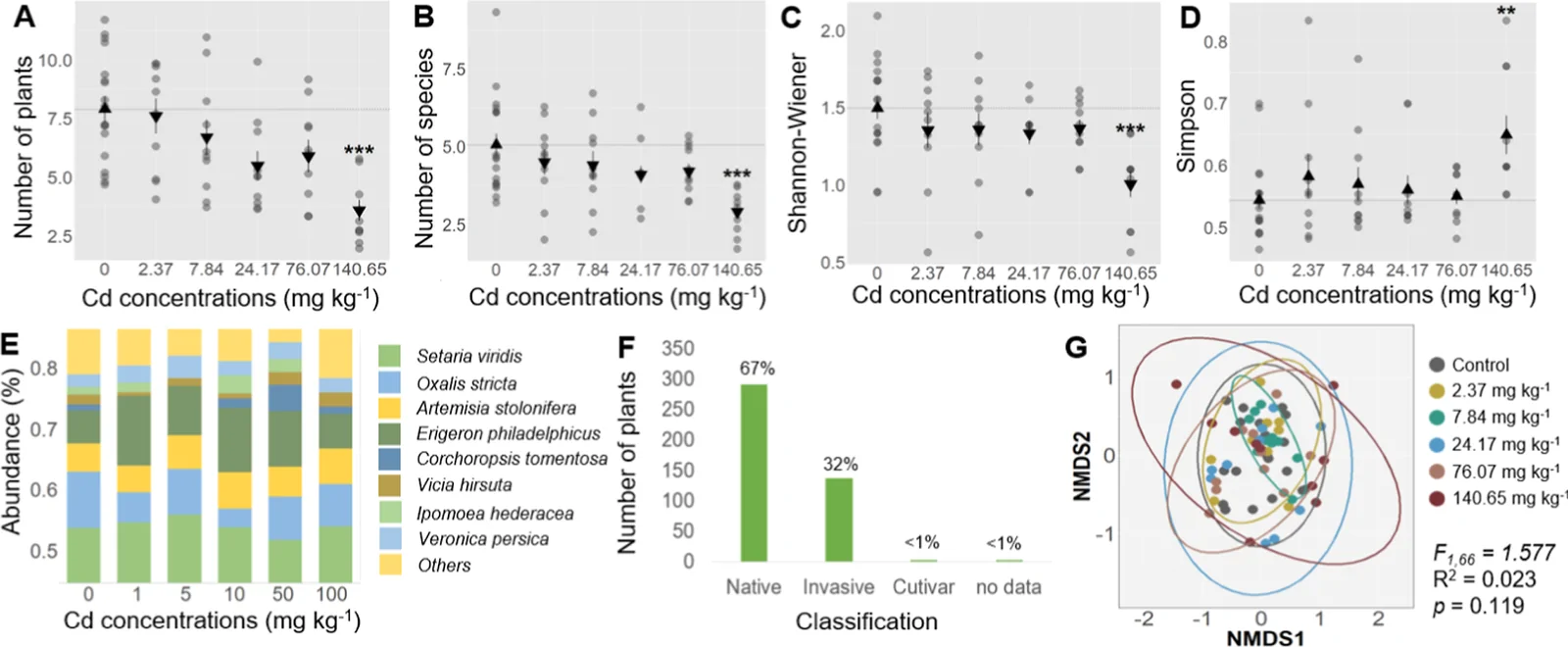
Impact of cadmium (Cd) exposure on plant community diversity and structure. (A) Total number of individuals per pot, (B) species richness (number of species), (C) Shannon diversity index, (D) Simpson dominance index, (E) relative abundance of the top eight dominant species, (F) species composition by category (native, invasive, and cultivars), and (G) nonmetric multidimensional scaling (NMDS) ordination based on Bray–Curtis dissimilarity. In panels (A–D), gray circles represent individual data points, black triangles indicate the mean, and error bars represent the standard deviation. Asterisks denote significant differences from the control group based on Dunnett’s test or Dunn’s test (*, *p* < 0.05; **, *p* < 0.01; ***, *p* < 0.001). In panel (G), each data point represents an individual pot, and the colored ellipses signify treatment groupings. Community structural shifts were assessed via PERMANOVA (*F*_1,66_ = 1.577, *R* ^2^ = 0.023, *p* = 0.119).

Cd is a nonessential element for plants, and its toxicity is well-known to induce growth abnormalities and inhibition in many agricultural species, such as *Cicer arietinum*, *Lens culinaris*, *Triticum aestivum*, and *Zea mays*.^34–36^ However, its impacts on community structure and diversity have seldom been prioritized as toxicological end points. Field surveys have revealed that metal-contaminated sites exhibit reduced plant species richness and diversity compared to uncontaminated areas, often selecting for metal-tolerant taxa.^37–39^ While these studies suggest that plant community diversity is primarily driven by soil metal contents^37^ or that high plant diversity mitigates soil contamination,^39^ it remains challenging to isolate specific environmental drivers from observed ecological effects. In the present study, we isolated the effect of a single variable (Cd addition) under controlled experimental conditions, including a robust reference set (control group). The effective concentration 10% (EC_10_) of Cd for species richness and the Shannon–Wiener index was calculated as 2.02 and 76.36 mg kg^–1^, respectively (Table S6), and these values were comparable to or up to approximately 20-fold higher than the hazardous concentration 5% (HC5, 0.59–3.57 mg kg^–1^) derived from conventional SSD models.^40^ These metrics differ in their conceptual basis and should not be directly compared. HC5 represents a conservative benchmark designed to protect 95% of various species including soil invertebrates (e.g., earthworms and springtails), based on conventional single-species toxicity data (EC_10_ or NOEC value).^41,42^ Conversely, our community-level EC_10_ reflects an empirical threshold incorporating interspecific interactions among plant species. Nevertheless, the observed gap between HC5 and community-level EC_10_ offers critical insights into the conservative margin of current regulatory standards.

Although the toxicity thresholds in our study showed wide confidence interval ratio (ranging from 8 to 112), which typically indicates low statistical precision,^42^ such variation is often unavoidable in community-level assessments, due to the wide spectrum of multispecies interactions between highly vulnerable and tolerant taxa.^43^ Regulatory guidance often recommends retaining only data that meet stringent statistical precision criteria.^41,42^ While such conservative filtering minimizes analytical noise in laboratory bioassays, applying these rigid cutoffs to community-level assessments may discard ecologically meaningful data. In community-level assessments, flexible approaches are strongly recommended to capture heterogeneous sensitivity patterns while maintaining protective benchmarks in ecological risk assessment.^44^ We emphasize that the community-level responses in our study reflect more realistic toxicological profiles than those provided by single-species assays.

### 3.2 Biomass Responses of the Plant Community

The growth of the plant community was markedly inhibited by Cd exposure, and a trend was clearly discernible upon visual examination (Figure 3A). Total shoot biomass (per pot) was significantly suppressed at the highest Cd concentration (140.65 mg kg^–1^) (Figure 3B). Notably, the top three dominant species exhibited no significant growth reduction across all treatments. The dominant species *S. viridis* showed a compensatory increase in shoot biomass at intermediate Cd concentration, and the shoot and root growth of *O. stricta* were significantly stimulated at intermediate levels (Figure 3B and C). The relationship between total shoot biomass (per pot) and alpha-diversity was slightly modulated by Cd concentration (Figure 4). At the lowest (2.37 mg kg^–1^) and the highest (140.65 mg kg^–1^) Cd levels, the Shannon–Wiener diversity exhibited a significant positive correlation with total shoot biomass (*R* ^2^ = 0.451 and 0.746, respectively; *p* < 0.05) (Figure 4A). Conversely, the Simpson index showed a significant negative correlation with biomass at the same concentrations (*R* ^2^ = 0.466 and 0.658, respectively; *p* < 0.05) (Figure 4B).

**Figure 3:**
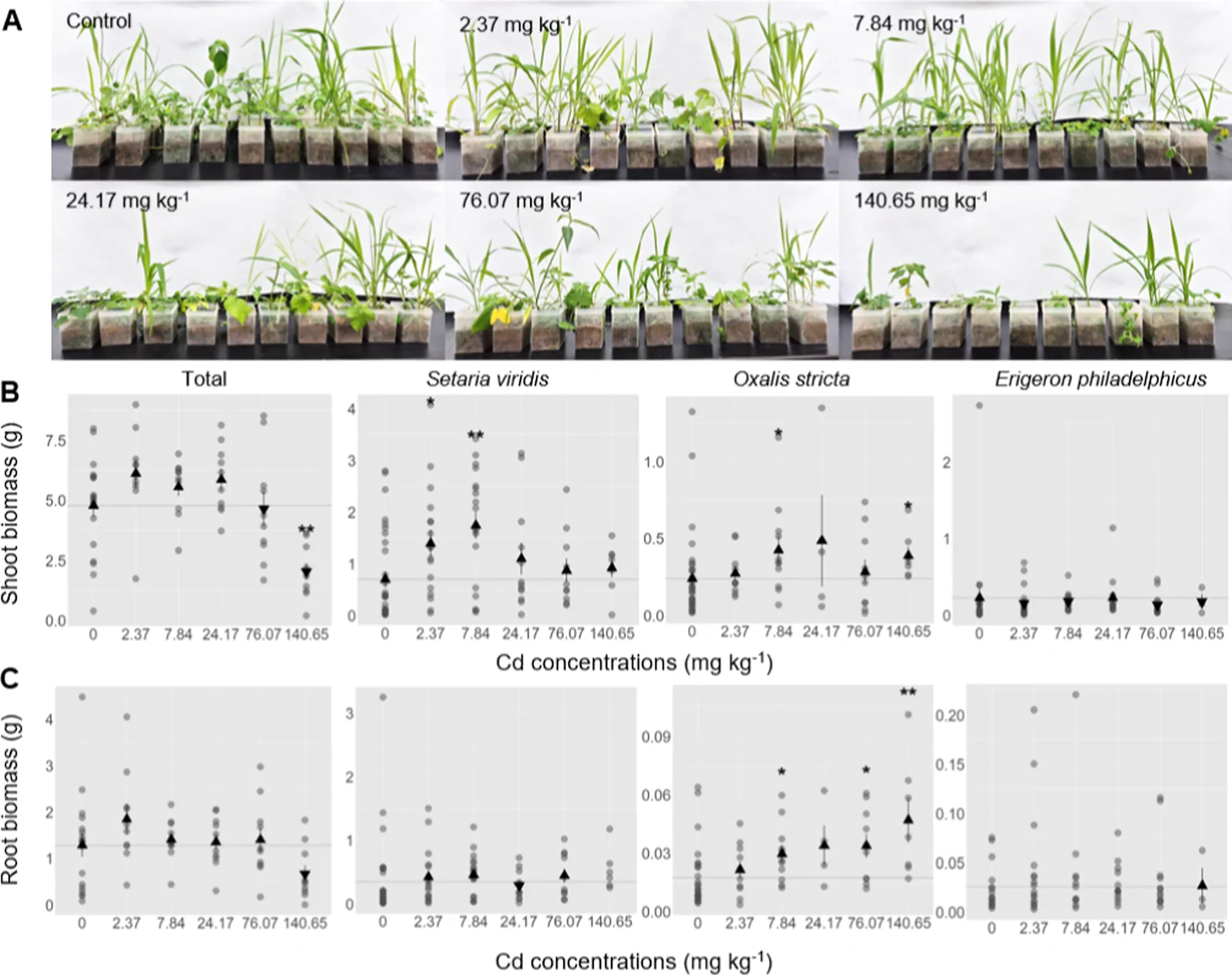
Phenotypic and biomass responses of the plant community and dominant species under cadmium (Cd) stress. (A) Representative photographs of experimental pots across the Cd concentration gradient, illustrating dose-dependent growth inhibition. (B) Shoot biomass and (C) root biomass for the total community and the three most dominant species (*Setaria viridis*, *Oxalis stricta*, and *Erigeron philadelphicus*). In panels (B–C), gray circles represent individual data points, black triangles indicate the mean, and error bars represent the standard deviation. Asterisks denote significant differences from the control group based on Dunnett’s test or Dunn’s test (*, *p* < 0.05; **, *p* < 0.01; ***, *p* < 0.001).

**Figure 4:**
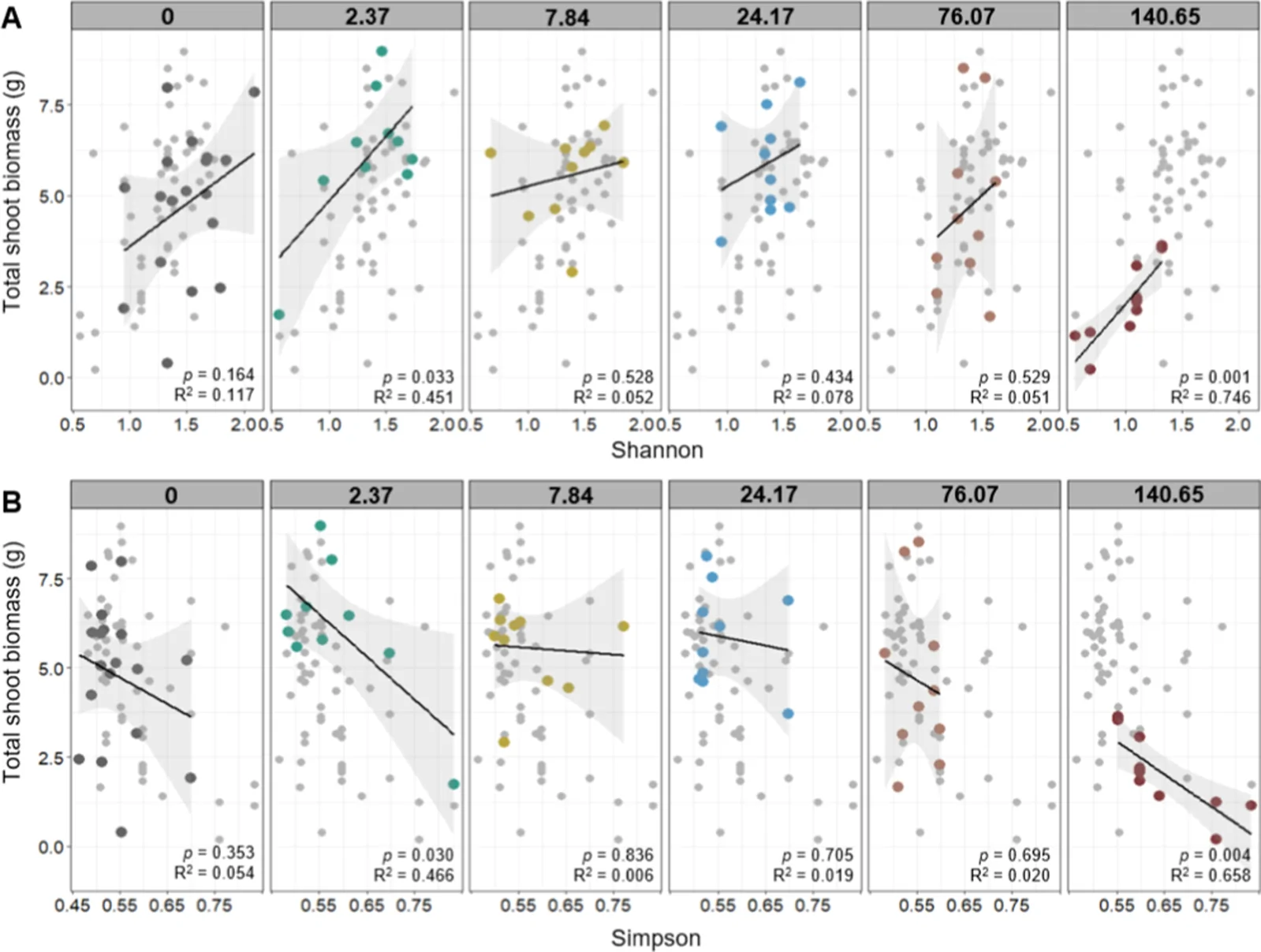
Correlation between community productivity and alpha-diversity across cadmium (Cd) concentrations. Relationships between the total shoot biomass per pot and diversity indices: (A) Shannon diversity index and (B) Simpson dominance index. Each data point represents an individual pot from the control and Cd treatment groups (mg kg^–1^). Pearson correlation coefficients and associated p-values are indicated for each relationship. Solid lines represent the linear regression fits, with shaded areas indicating the 95% confidence intervals.

Despite a significant reduction in total shoot biomass (EC_50_: 117.87 mg kg^–1^) (Table S6), the dominant species showed no significant changes even at the highest Cd concentrations. Given that EC_50_ for shoot growth in agricultural species typically ranges from 61 to 268 mg kg^–1^,^34^ the dominant species in our study (*S. viridis*, *O. stricta*, and *E. philadelphicus*) exhibited robust tolerance to Cd toxicity.^45,46^ This suggests that the effects on the community level were driven by the selective loss of Cd-sensitive species rather than a uniform inhibition across all constituents. The correlation analysis between biomass and alpha-diversity further demonstrated that community productivity (biomass) is maximized when diversity (Shannon–Wiener) and dominance (Simpson) is high. While these correlations weakened under intermediate levels, they became most pronounced in the highest Cd concentration. This trend implies that as the community simplified, the surviving species maintained strong functional interactions.^15^ This hypothesis is supported by our observation that root biomass remained stable despite a significant decline in species richness. Although Cd stress typically inhibits root growth by reducing cell wall elasticity,^47^ the surviving species in our system appeared to be resistant to these stressors, potentially benefiting from competitive release and interspecific interactions.^48^ For instance, *O. stricta* is well-known as a noncompetitive species and easily suppressed by dense ground cover.^49^ However, it also possesses a remarkable tolerance to metal toxicity,^45^ and in our study, it manifested significant root stimulation at the highest Cd concentration. This result suggests that for certain tolerant species, the ecological benefits gained from the competitive release following the loss of vulnerable neighbors may outweigh the effects of metal stress.

### 3.3 Co-Occurrence Network Analysis

In the control group, the plant community formed a highly interconnected network consisting of 21 species with 16 active interactions (edges). However, under Cd exposure, the number of active nodes (species with valid interactions) decreased to 12–14, with the number of edges ranging from 3 to 6 (Figure 5 and Table S7). To control for potential bias by variations in node count, a rarefaction analysis was performed by subsampling 12 nodes (minimum number of nodes) over 1000 iterations. Specifically, the rarefied average strength and modularity of the control group dropped from 0.880 to 0.479 and from 0.477 to 0.339, respectively (Table S7). These corrections demonstrate that the network strength and modularity initially observed in the control data were largely affected by species richness. Conversely, the mean edge weight remained consistently higher across all treatments (0.635–0.668) relative to the control network (0.580). This persistent elevation indicates that while toxic stress reduced overall species richness (node loss), the interspecific co-occurrence strength among surviving species became intensified. These metrics collectively indicate a transition toward a more condensed and tightly coupled network architecture under chemical stress.

**Figure 5:**
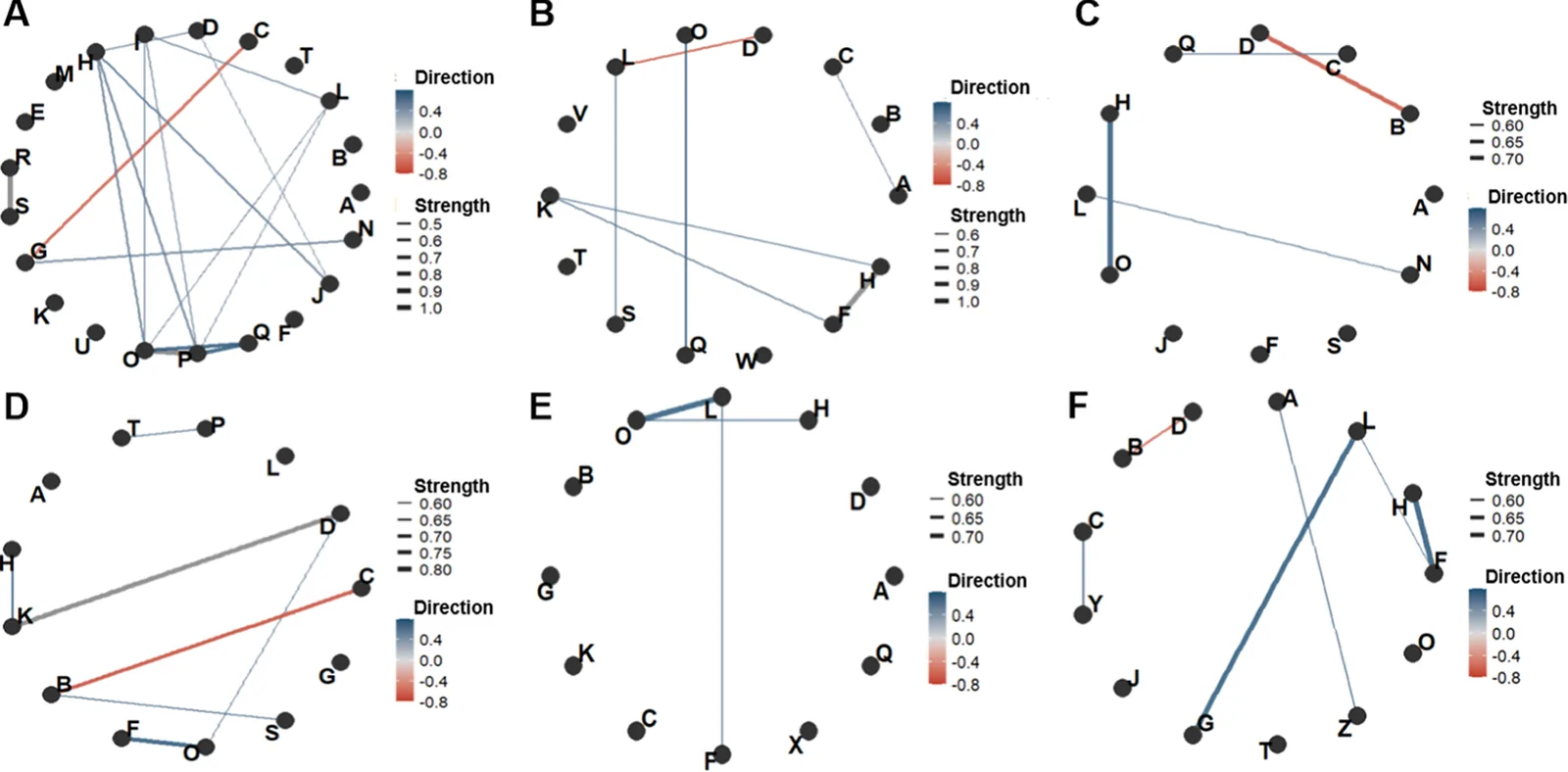
Co-occurrence network analysis of plant community according to each treatment. (A) 0, (B) 2.37, (C) 7.84, (D) 24.17, (E) 76.07, and (F) 140.65 mg kg^–1^. Line colors indicate the direction of interactions (blue for positive, red for negative correlations), and line thickness represents the interaction strength (correlation coefficient). Each species label (A to Z) corresponds to the species information provided in Table S2, and edge density, mean edge weight (interspecific association strength), average strength (overall node connectedness), and modularity provided in Table S7.

Our results reveal that Cd stress acts as a stringent environmental filter, forcing the plant community into a more simplified network (node loss) alongside strong interspecific interactions (higher mean edge weight). These trends indicate that environmental stress clusters surviving species into a synchronized response profile.^50^ Under severe toxicity, the specialized interactions that define distinct modules are superseded by a universal stress factor, leading to a structurally homogenized network where niche differentiation is eroded.^51^ Overall, our findings suggest that environmental stress erodes the structural complexity of plant communities, driving resilient species into patterns of elevated network strength.

### 3.4 Potentials and Limitations

The primary objective of this study was to validate an experimental approach using soil seed banks as a robust ecotoxicological tool for assessing community-level responses. Our findings demonstrate the efficacy of this methodology through three advancements: (1) we successfully transitioned from single-species bioassays to a rapid and holistic assessment of field-derived plant communities; (2) we established the utility of community-level parameters as informative toxicological end points; and (3) we ensured high ecological realism while maintaining laboratory-scale reproducibility and repeatability, effectively bridging the gap between controlled laboratory conditions and complex field dynamics. Despite these advancements, several limitations remain to be addressed in subsequent research: (1) while Cd served as an effective model contaminant, a broader spectrum of chemical stressors (e.g., organic pollutants, microplastics, or pesticides) is necessary; (2) although structural changes in the community were evident, biochemical and molecular profiling (e.g., metabolomics) would further elucidate the precise mechanisms underlying interspecific toxicological responses; and (3) future models should incorporate perennial species and other trophic levels, such as microorganisms or soil fauna, to achieve a more comprehensive understanding of ecosystem functions.

Ecosystems are not merely an assembly of individual species; they maintain stability against external stressors through intricate interspecific networks. The biodiversity and reciprocal networks found in nature serve as a robust survival strategy and a cornerstone for evolutionary adaptation. In this study, we addressed a major hurdle in ecotoxicology by successfully bridging the gap between field-scale ecological realism and controlled laboratory experimentation. Our approach provides direct evidence of toxicological impacts at the community level. We believe the fundamental utility and reliability of this seed bank-mediated approach was firmly validated. To overcome the limitations of this study, future research should incorporate diverse substances and broader field sampling to enhance reproducibility and applicability. We anticipate that the continued refinement of this method will catalyze the development of next-generation ecological risk assessments, facilitating a deeper understanding of anthropogenic impacts on terrestrial ecosystems.

## Supplementary Material



## Notes

Tables S1–S7 summarizing soil physicochemical properties, heavy metal concentrations, plant species list, statistical diagnostic test results, raw biomass and diversity data sets, calculated toxicity effect concentrations, network parameters, and rarefied null model estimates (XLSX)
